# Compressive spectral image fusion via a single aperture high throughput imaging system

**DOI:** 10.1038/s41598-021-89788-y

**Published:** 2021-05-13

**Authors:** Hoover Rueda-Chacon, Fernando Rojas, Henry Arguello

**Affiliations:** Department of Computer Science, Universidad Industrial de Santander, Bucaramanga, 680002 Colombia

**Keywords:** Electrical and electronic engineering, Computer science, Optical sensors, Imaging techniques, Spectrophotometry

## Abstract

Spectral image fusion techniques combine the detailed spatial information of a multispectral (MS) image and the rich spectral information of a hyperspectral (HS) image into a high-spatial and high-spectral resolution image. Due to the data deluge entailed by such images, new imaging modalities have exploited their intrinsic correlations in such a way that, a computational algorithm can fuse them from few multiplexed linear projections. The latter has been coined compressive spectral image fusion. State-of-the-art research work have focused mainly on the algorithmic part, simulating instrumentation characteristics and assuming independently registered sensors to conduct compressed MS and HS imaging. In this manuscript, we report on the construction of a unified computational imaging framework that includes a proof-of-concept optical testbed to simultaneously acquire MS and HS compressed projections, and an alternating direction method of multipliers algorithm to reconstruct high-spatial and high-spectral resolution images from the fused compressed measurements. The testbed employs a digital micro-mirror device (DMD) to encode and split the input light towards two compressive imaging arms, which collect MS and HS measurements, respectively. This strategy entails full light throughput sensing since no light is thrown away by the coding process. Further, different resolutions can be dynamically tested by binning the DMD and sensors pixels. Real spectral responses and optical characteristics of the employed equipment are obtained through a per-pixel point spread function calibration approach to enable accurate compressed image fusion performance. The proposed framework is demonstrated through real experiments within the visible spectral range using as few as 5% of the data.

## Introduction

Hyperspectral imaging technology has been introduced in the imaging market mainly within remote sensing devices, due to its capabilities to detect and classify minerals, vegetation, man-made materials, and others^1–3^. This technology has been also used in medicine to conduct guided surgery and anomaly detection^4–6^. The design of the electro-optical sensor systems used in hyperspectral imaging often has to deal with the fundamental trade-off between spatial and spectral resolution, as well as the light throughput required to attain good signal-to-noise-ratio (SNR) in the acquired image^7,8^. More specifically, conventional hyperspectral image sensors employ an aperture/slit which controls the amount of light allowed into the imaging system, and a collection of optical elements such as mirrors and a dispersive element to decompose the light into multiple wavelengths. Smaller slits yield to better spectral resolution, but poorer light throughput and vice versa. Further, the light throughput of the system is affected by the employed optical elements, and the intensity manipulation inside the imager determines the corresponding resolution. For instance, panchromatic systems, which do not employ dispersive elements, and thus, integrate light intensity along several hundreds of nanometer bandwidths, offer far better SNR and spatial resolution than multispectral (MS, tens of bands) or hyperspectral (HS, hundreds of bands) systems. Conversely, HS systems offer richer spectral information than MS and panchromatic systems, at the cost of lower SNR in the sensor output. In order to have high spatial and spectral resolution, remote sensing platforms incorporate multiple sensors with concurrent capabilities so as to capture hyperspectral imagery along with panchromatic or multispectral imagery of higher spatial resolution^7^, which are then post-processed to attain the best characteristics of both worlds through image fusion techniques^9^. In particular, the fusion of HS and MS images has been previously demonstrated^10–12^, where fused data exhibits the spectral characteristics of the observed HS image (i.e., spectral sensitivity and photon efficiency) at the spatial resolution and sampling of the MS image (i.e., spatial detail and texture structure), which contributes to the accurate identification and classification of an area at a fine spatial resolution. Nonetheless, conventional spectral imaging devices have the drawback of requiring to scan a number of zones that grows linearly in proportion to the desired spatial or spectral resolution^13,14^.

Due to the data deluge entailed by spectral images, new imaging modalities have been proposed to exploit their intrinsic correlations to sample and compress (mix or multiplex) them, in such a way, that a computational algorithm can recover (unmix or demultiplex) such data from few linear projections, which makes them attractive for many practical applications. The latter has been called compressive spectral imaging (CSI) framework^15–18^. In particular, CSI multiplexed projections are obtained by encoding, via spatial light modulators (SLMs), shearing, via dispersive elements, and integrating the spectral information along the spatial extent of a monochrome image sensor^15–20^ or a single pixel detector^21,22^. Multiplexed data are then processed by a computational algorithm that solves an inverse problem to estimate the full spectral image relying on sparsity, low-rank or deep learned priors. A vast amount of works have been proposed in CSI, most of them aiming to find and implement the best optical encoding and processing protocols that lead to good multiplexed measurements, and to relax the ill-posedness of the inverse problem, so as to improve the reconstruction image quality. Some of these works have proposed the usage of multiple-snapshots^16–18^, varying the coding pattern used in the SLM, or the usage of a secondary panchromatic^23,24^ or color^25^ sensor to guide the image reconstruction, either through a dictionary-training step or as prior-information for a maximum-a-posteriori algorithm. In contrast, given that information from a secondary panchromatic or color sensor falls short in terms of spectral richness, more recent works have proposed to conduct spectral image fusion from MS and HS compressed measurements^26–29^. In general, fusion approaches have provided better results, in terms of image quality and resolution enhancement, but they have focused mainly on the algorithmic part, simulating the behavior of optical instrumentation. Therefore, none of them, to the authors’ knowledge, have demonstrated the viability of compressed spectral image fusion through a real optical prototype.

**Figure 1:**
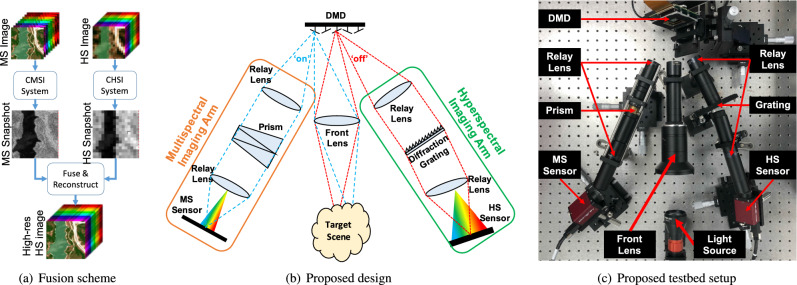
Proposed compressive spectral image fusion (CSIF) system. (**a**) Snapshots are acquired with a compressive MS imager (CMSI) and a compressive HS imager (CHSI), which are then fused and reconstructed via a computational algorithm. (**b**) Sketch of the proposed design, and (**c**) Proposed testbed setup. The front lens images the target scene onto the DMD, which encodes and reflects the scene towards $\pm 24^{\circ }$ (on/off), where the MS and HS imaging arms are located. Both imaging arms employ 4F relay lenses to transmit the encoded light to the sensors, either through the prism, or through a grating.

Motivated by this, in this manuscript we propose a unified computational imaging framework that includes the design and implementation of a proof-of-concept optical architecture (or testbed) to demonstrate the viability of compressive spectral image fusion, along with the formulation of a convex algorithm to solve the inverse problem that fuses the characteristics of the multispectral and hyperspectral compressed projections to estimate a spectral image with high spatial and high spectral resolution. A sketch of the compressive spectral image fusion (CSIF) scheme is shown in Fig. 1a. In particular, compressed snapshots are acquired via a compressive multispectral imager (CMSI) and a compressive hyperspectral imager (CHSI), and are then fused and used to estimate the high resolution data cube with the spatial details provided by the MS snapshot and the spectral details provided by the HS snapshot. The testbed relies on a digital micro-mirror device (DMD) to encode and split the incoming light intensity into two independent imaging arms, one for MS compressed projections and the other for HS compressed projections. We take advantage of the reflecting capabilities of the DMD to attain full light throughput sensing, using a single common aperture, since the coded light goes towards one of the arms and its coded complement goes through the second arm. To combat the optical path length difference (OPLD) generated by the micromirrors flipping angles, and thus to obtain sharp images in both imaging arms, we rely on the Scheimpflug compensation. Further, we exploit the dynamically changing capability of the DMD to attain multiple snapshots with different coding patterns. The testbed construction includes the characterization of the real spectral responses and optical characteristics of the employed equipment, which are calibrated to guarantee accurate image fusion. The fusion+reconstruction algorithm is built following the alternating direction method of multipliers (ADMM) methodology to solve a convex optimization problem that imposes sparsity priors and exploits the Sherman–Morrison–Woodbury inversion lemma to speed up the estimation process. In summary, the main contributions of this paper are two-fold: First, the design and construction of the optical testbed imaging system, with full-throughput sensing capability; and second, the formulation of a convex optimization problem to fuse and reconstruct the spectral cube from the compressed measurements attained with the testbed.

## Results

### Compressive spectral image fusion: acquisition scheme

State-of-the-art works have proposed the fusion of HS and MS images from compressed measurements^26–29^. These works assume that the light intensity from the target scene splits into two imaging systems, which independently encode the scene with a spatial light modulator (SLM) or digital micromirror device (DMD), and then disperse the light via a prism or diffraction grating to attain the compressive MS and HS measurements. Further, these systems assume a perfect match between the DMD pixel size and those of the sensors. Thus, since a MS sensor has high spatial resolution, the DMD used in the MS system also exhibits such resolution, whereas the HS sensor exhibits fewer pixels but of greater size. Having two independent systems, attaining the MS and HS compressed measurements, implies a critical loss in terms of light throughput, since the coding patterns would prevent a certain portion of the incoming light from reaching the sensors. Thus, since two independent encoding devices are assumed, double light loss will occur.

In contrast, the proposed imaging system, shown in Fig. 1b, overcomes the throughput issue by exploiting the reflecting capabilities of the DMD, such that when the mirrors are in the ‘on’ state, the encoded light goes through one of the arms, and its complement (light from the mirrors that are in the ‘off’ state), which is also encoded, goes through the second arm, thus attaining full light throughput (50% in each sensor). In particular, the DMD is placed in the image plane of the front lens, which images the target scene. A coding pattern is loaded in the DMD, with half the entries letting the light to pass, and the other half blocking it. Remark that entries letting the light to pass will reflect towards the + 24$^\circ$ angle, whereas the light being blocked will reflect towards the $-24^\circ$ angle. Therefore, we place the MS imaging arm at the $+24^\circ$ path and the HS imaging arm at the $-24^\circ$ path. The MS imaging arm is composed of a relay lens, a prism and a high-resolution image sensor (MS sensor). Similarly, the HS imaging arm comprises a relay lens, a diffraction grating and a low-resolution image sensor (HS sensor). Remark that the differences between both imaging arms lie in that the grating entails greater dispersion than the prism, and the MS sensor exhibits better spatial resolution than the HS sensor.

The proposed imaging system presents some interesting challenges. First, if the resolution of the DMD is set to match the high spatial resolution of the MS sensor, the HS imaging arm will work with a down-sampled coding pattern; on the contrary, if the DMD is set to match the low spatial resolution of the HS sensor, the MS imaging arm will work with a replicated coding pattern, thus losing spatial details. The second challenge relates to the alignment of both imaging arms with the DMD, so that, sharp images are integrated in both sensors. This challenge arises because the rotation axis of each micro-mirror works along the surface diagonal and the ‘on’ and ‘off’ tilting angles induce an optical path length difference which, in turn, causes non-uniform focus and distortion in the image sensors. The first challenge can be solved by either multi-frame sensing, exploiting the rapid DMD refresh frequency rate and synchronizing the cameras so that in one frame the DMD shows a coding pattern that matches the MS sensor resolution and the consecutive coding pattern is binned to match the HS sensor resolution, or by correctly modeling the down-sampled pattern in the forward (Eqs. (1)–(8)) and inverse model (Eqs. (9)–(14)). The second challenge can be solved by first rotating the DMD by 45$^\circ$ with respect to the axis perpendicular to the plane of the micro-mirrors, such that the rotation axis of each mirror coincides with the vertical, hence resulting in a light path along the horizontal plane; then, to solve the optical path length difference, the Scheimpflug principle must be considered to capture all-in-focus images in both image sensors^30–32^.

#### Scheimpflug compensation

**Figure 2:**
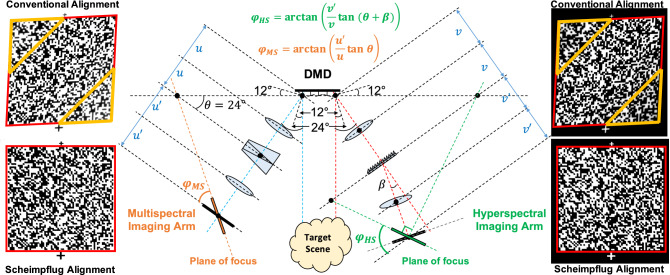
Scheimpflug alignment. Given the $\pm 12^{\circ }$ inclination of the DMD micro-mirrors, light perpendicularly impinging the DMD reflects at $\pm 24^{\circ }$. This causes non-uniform focus (within the yellow triangles, in the conventional alignment insets) and distortion of the image in the sensor (inclination), due to the different optical path lengths. To correct this, the sensors must be rotated along the horizontal axis by the Scheimpflug angles $\varphi _{MS}$ and $\varphi _{HS}$, respectively. The latter depends on the angle ($\beta$) of the first diffraction order. Note that the images in both sensors are complementary to each other.

One of the main challenges of the proposed CSIF system is the correct alignment of both imaging arms with the ‘on’ and ‘off’ reflecting angles of the DMD. First, as mentioned above, each micro-mirror on the DMD rotates, by default, with respect to its surface diagonal. Then, if the vertical axis of the DMD is placed normal to the horizontal plane, the micro-mirrors will reflect the incident light out of the horizontal plane along 45$^\circ$^33^. Therefore, to facilitate the optical alignment, the DMD is rotated 45$^\circ$ with respect to its perpendicular axis to align the rotation axis of the micro-mirrors with the vertical axis. This in turn, will translate the light propagation workplace from the 45$^\circ$ plane to the horizontal axis. Second, since the micro-mirrors tilt to $\pm 12^{\circ }$ by default^33^, light perpendicularly impinging the DMD reflects towards $\pm 24^{\circ }$, respectively, following the principle of total reflection, as illustrated in Fig. 2.

Analyzing the MS imaging arm in Fig. 2, note that if the image sensor is placed normal to the $+24^{\circ }$ optical axis, an image with a non-uniform focus and magnification will be attained due to the difference in path lengths of the rays reflected by the DMD. Section S1 of the supplementary material provides more technical details related to the optical path length difference. This occurs because the micro-mirrors exhibit an inclination of 12$^{\circ }$, and light from the target scene hits them non-uniformly. To compensate for the tilting angle of the micro-mirrors, the image sensor must be tilted contrarily by an angle $\varphi$, which has been coined in the literature as the Scheimpflug angle^30–32^. The principle behind Scheimpflug compensation is that the plane of focus is given by the projection of the line that intersects with the DMD normal plane and the lens plane, as detailed in the left half of Fig. 2. In particular, for the MS imaging arm, and given that a 4F relay lens is used, the angle can be calculated as $\varphi _{MS} = \arctan \left( (u'/u) \tan \theta \right)$, where *u* and $u'$ are the distances from the DMD to the lens, and from the lens to the sensor, respectively^31^. Note that since we use an Amici prism^16^, the light dispersion goes straight to the sensor. Regarding the HS imaging arm, the 4F-relay is also considered to behave as a single lens, with a focal distance *v*. However, since a diffraction grating is used, light is dispersed onto different orders following its blaze angle, $\beta$. Therefore, the Scheimpflug angle is calculated with respect to the normal plane of the first diffraction order, which is the one used in this work. That is, the Scheimpflug angle for the HS arm is given by $\varphi _{HS}=\arctan \left( (v'/v) \tan (\theta +\beta ) \right)$, as detailed in the right half of Fig. 2. Examples of the images captured in both image sensors, following the conventional alignment (without Scheimpflug) and after Scheimpflug compensation, are shown on both sides of Fig. 2. Note that in the conventional alignment, the attained images are out of focus (within the yellow triangles) and distorted (along the x–y axis), but they are corrected after Scheimpflug compensation.

### Experimental testbed

#### Proof-of-concept implementation and alignment

To test the proposed imaging framework, we built a proof-of-concept optical prototype, shown in Fig. 1c and detailed in Section S2 of the supplementary material, which employs an objective lens (Tamron, 8mm 1.1”) paired with a relay lens (Thorlabs, MAP10100100-A) to image the target scene onto a DMD (Texas Instruments, DLI4130VIS-7XGA) with a micro-mirror size of $13.68 \upmu m$, that encodes the incoming light. Both imaging arms use 4F-relay systems built using two lenses (Thorlabs, AC254-100-A-ML) to transmit the encoded light through the dispersive elements placed at the Fourier plane. The MS imaging arm employs a dual Amici prism (Shanghai Optics, custom made) with central wavelength 550 nm, whereas the HS arm uses a transmission diffraction grating (Thorlabs, GT50-03, 300 grooves/mm, 17.5$^{\circ }$ groove angle). Regarding the image sensors, both arms employ equal monochrome sensors (AVT, Stingray F-145B, working at 14 bits) with $1392 \times 1040$ pixels and a pixel size of $6.45\times 6.45 \upmu m$. To emulate a low-resolution sensor, $2\times 2$ pixel binning was performed in the sensor sitting at the end of the HS arm, thus attaining a $696 \times 520$ image sensor with a pixel size of $12.9\times 12.9 \upmu m$.

To correctly align the two imaging arms with the DMD ‘on’ and ‘off’ reflection angles, a monochromatic collimated light beam, obtained by attaching an achromatic Galilean beam expander (Thorlabs, GBE05-A) to a tunable Xenon Arc Light source (Oriel Instruments TLS-300XR, with a 200 $\upmu m$ slit) was shined perpendicularly to the DMD plane, without the objective lens in place. Recall that to facilitate the optical alignment, the DMD was rotated by 45$^{\circ }$ with respect to the optical axis. Knowing that the normal of the DMD micro-mirrors tilts $\pm 12^{\circ }$, the relay lenses were placed at $\pm 24^{\circ }$, respectively. Then, the image sensors were also rotated by 45$^{\circ }$, to match the rotation of the DMD, and were placed behind the relay lenses to obtain a sharp image of the DMD micro-mirrors in both arms. These sharp images roughly satisfy a 1:4 correspondence between the DMD and the MS sensor pixels, and a 1:2 correspondence between the DMD and the HS sensor pixels. Afterwards, the prism was placed in the MS arm, also rotated at 45$^{\circ }$, and the MS sensor was slightly shifted to account for the focal length shift of the prism, thus guaranteeing the match between the DMD and sensor pixels. Similarly, on the HS arm, the grating was rotated and placed in between the 4F relay system, and the second relay lens and the HS sensor were tilted-and-shifted accordingly, to match the first diffraction order of the grating. Remark that a monochromatic beam, ranging from 480 to 650 nm, was used at all times during the alignment to guarantee the dispersion of both dispersive elements along the horizontal axis.

At this point, the images in each sensor resemble those termed “Conventional Alignment” in Fig. 2, when a random pattern is loaded in the DMD and illuminated at 650 nm. By careful analysis, we can observe that these images exhibit blurred zones along the edges, highlighted with yellow triangles, and a slight distortion (inclination) along the $x-y$ axis, due to the optical path difference caused by the micro-mirrors reflection angle. To alleviate the non-uniform focus and distortion, both image sensors were rotated by their corresponding Scheimpflug angles, $\varphi _{MS}$ and $\varphi _{HS}$, along the horizontal (propagation) axis, such that the images in each sensor resemble those termed “Scheimpflug Alignment” in Fig. 2. In particular for the MS arm, the 4F relay lens has an effective focal length of 100 mm; thus, $u = 100$ mm and $u' = 100 + f_p$ (mm), where $f_p$ is the focal length entailed by the prism. Note that although the prism does not have an effective focal length, the prism itself yields a slight focal shift, $f_p \approx 2.5$ mm (calculated experimentally). Therefore, $\varphi _{MS} \approx 22.97 ^\circ$. Similarly, for the HS arm, the 4F relay employs two lenses with effective focal length of 100 mm; thus, $v = 100$ mm and $v' = 100$ mm. Since the first diffraction order of the grating occurs at exactly 17.5$^{\circ }$, $\varphi _{HS} \approx 41.5 ^\circ$. After both imaging arms are aligned, the front lens is placed instead of the collimated beam, as shown in Fig. 1c, to conduct image formation of the target scene onto the DMD.

#### Testbed calibration

**Figure 3:**
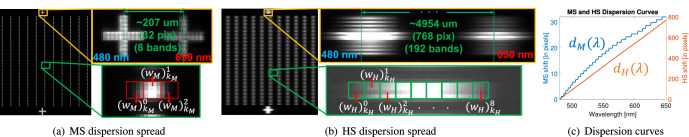
Characterization of dispersion functions. (**a**) Image obtained in the MS arm at 550 nm. (**b**) Image obtained in the HS arm at 550 nm. (**c**) Non-linear MS dispersion curve (left y-axis) and linear HS dispersion curve (right y-axis) in terms of wavelength. The prism spreads the 480–650 nm spectral range along 207 $\upmu m$ (up to 32 MS sensor pixels), whereas the diffraction grating disperses it along 4954 $\upmu m$ (up to 768 HS sensor pixels). Each DMD pixel affects three MS sensor pixels, with intensity weights $(w_M)_{k_M}^0, (w_M)_{k_M}^1, (w_M)_{k_M}^2$ (as detailed in Table S1 in the supplementary material), while the same DMD pixel impinges in up to 9 HS sensor pixels with intensity weights $(w_H)_{k_H}^0, (w_H)_{k_H}^1, \ldots , (w_H)_{k_H}^8$ (as detailed in Table S2 in the supplementary material). $k_M$ indexes the MS bands and $k_H$ indexes the HS bands, as explained in the “Methods” section.

To calibrate the system impulse response, each sensor pixel was characterized to reduce the imperfections entailed by optical aberrations, radiance signal strength, DMD filtering, and pixel mismatch between the sensor and the DMD. To this end, and given the non-ideal responses of the employed dispersive elements, the higher-order propagation modeling^34^ was used to account for a better approximation of the light propagation through the proposed system. In particular, a set of coded apertures was first designed satisfying that, (1) all pixels in the active area of the DMD must be active at least and only once, (2) no multiplexing occurs in the sensor, (3) the number of patterns is minimized. Note that the third constraint is critical, since the trivial but worst-case scenario uses the same number of patterns as pixels, each one with a single active pixel. For our system, given the high dispersion caused by the diffraction grating of the HS arm, as few as 24 patterns, of size $256\times 256$, are required to satisfy the three restrictions above. These 24 patterns are cyclic horizontal and vertical permutations of a pattern with equally-spaced ‘on’ pixels, similar to a checker pattern, but with more ‘off’ space in between, so as to avoid spectral multiplexing in the sensor due to the dispersion. An example of one of these patterns can be appreciated in the leftmost insets of Fig. 3a and b.

These patterns were loaded into the DMD, one at a time, while the system imaged a white calibration target illuminated with monochromatic light from the tunable light source varying the wavelength from 480 to 650 nm, in 0.5 nm steps. Ten frames were acquired and averaged at each wavelength to reduce the impact of shot noise. Further, we calibrated for temperature, readout noise, and death pixels, by placing a blocking-cap to the front-lens, turning off the light source and measuring a black reference image which was then subtracted from the calibration cubes. Given the 1:4 and 1:2 pixel correspondences between the DMD and the MS and HS sensors, respectively, the resulting calibration cubes exhibit $1024\times 1024$ spatial pixels and 341 wavelengths (from 480 to 650, in half nm steps) for the MS sensor and $512\times 512\times 341$ for the HS sensor.

The calibration cubes were used to estimate the dispersion curves of the prism and grating by binarizing the cross-mark, placed on top of the patterns, for each of the 341 wavelengths, and then measuring their centroid (x,y) location, as shown in Fig. 3a and b. The shifting x-coordinate was then plotted against wavelength, for each dispersive element, and the resultant curves are shown in Fig. 3c. Note that the total pixel shifts between the 480 nm and the 650 nm wavelengths are approximately $207 \upmu m$ and $4954 \upmu m$ for the prism and grating, respectively, which translates to 32 pixels of the MS sensor, and 768 pixels of the HS sensor. Further, Fig. 3c shows that the prism exhibits a non-linear behavior (left y-axis), whereas the grating entails a linear dispersion (right y-axis). To have a 1:1 pixel correspondence between the DMD and the sensors, the calibration cubes were down-sampled four times to obtain cubes of size $256\times 256\times 8$ for the MS arm and $128\times 128\times 192$ for the HS arm. Note that with this down-sampling, the bandwidth of each MS band is roughly $\sim 21$ nm, whereas that of the HS bands is approximately $\sim 0.89$ nm. Remark the huge spectral resolution difference between both spectral sensors.

Finally, the distribution of light hitting each sensor pixel was approximated to the real phenomena to guarantee a good image fusion quality^34^. For this purpose, the light distribution from each spectral band impinging onto the MS and HS sensors was calculated. For the MS arm, each multispectral band impinges up to 3 sensor pixels, with weights $(w_M)^{r_M}_{k_M}$, for $r_M=0,1,2$ and $k_M=0,1,\ldots ,7$, as depicted in Fig. 3a. For the HS arm, each hyperspectral band spreads along 9 sensor pixels with weights $(w_H)^{r_H}_{k_H}$, for $r_H=0,1,\ldots ,8$ and $k_H=0,1,\ldots ,191$, as depicted in Fig. 3b. Note that $r_M$ and $r_H$ index the MS and HS sensor pixels where the light intensity spreads. Therefore, three $256 \times 256 \times 8$ weight calibration data cubes for the MS arm, and nine $128 \times 128 \times 192$ weight calibration data cubes for the HS arm were obtained. By averaging each weight data cube per band, thus assuming a spatial-invariant system, succinct versions of the weights distribution can be estimated as shown in Tables S1 and S2 in Section S3 of the supplementary material. Note that these weight distributions, which sum-up-to-one, are the real entries of the sensing matrices ${\mathbf {H}}_M$ and ${\mathbf {H}}_H$ in Eq. 8.

#### Reconstruction results

**Figure 4:**
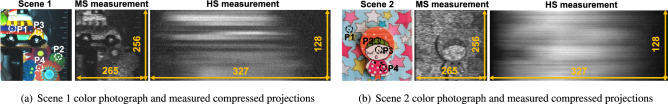
Target scenes and compressed measurements. Two target scenes are used to test the capabilities of the proposed system. (**a**) Scene 1. (**b**) Scene 2. The MS compressed measurements have size $256 \times 265$, whereas the HS compressed measurements have size $128 \times 327$. Spectral regions of interest are highlighted as P1–P4 in both color photographs.

To test the proposed testbed capabilities, two different target scenes, shown in Fig. 4, were used. The scenes were illuminated with a broadband halogen light source (Illumination Technologies, 3900E-ER), emulating ambient light conditions. The decimation ratios between the MS and HS measurements were $\Delta =2$ for the spatial resolution, since the MS measurements exhibit 256 rows and the HS measurements only 128, and $\Delta =24$ for the spectral resolution, since the MS measurements are the result of the dispersion of $L_M=8$ spectral bands and the HS measurements can resolve up to $L_H = 192$ bands.

To reconstruct the $256\times 256\times 192$ fused data cube, we employed the ADMM-based optimization algorithm, described in the “Methods” section, and evaluated the usage of $K=1$, $K=2$, and up to $K=4$ different snapshots, varying the coding pattern loaded in the DMD. An increasing number of snapshots reduces the compression ratio (*C*), and thus improves the conditioning of the inverse problem, which translates to better image quality. Nonetheless, the main goal of compressive imaging is to keep the number of snapshots at the smallest for a certain image quality. For our experiments, $K=1$ results in $C\approx 99\%$, $K=2$ in $C\approx 98\%$, and $K=4$ in $C\approx 96\%$. Thus, only $\approx 1\%$, $\approx 2\%$ and $\approx 4\%$ of the full spatial-spectral resolution data cube was used to recover it. For simplicity, the coded apertures were designed as realizations of a Bernoulli random variable, with a transmittance of 50%, matching the resolution of the MS sensor. Nonetheless, it should be noted that different methodologies^16,18^ can be employed to obtain optimized coded apertures to further improve image quality. However, the impact of these kinds of apertures lies out of the scope of this manuscript. Future work will undoubtedly look into it. The sparsity-promoting basis ($\varvec {\Psi }$) used in the ADMM algorithm, was set as the Kronecker product between the 2D Symlet 8 Wavelet transform $\varvec {\Psi }_{W2D}$ and the discrete cosine transform $\varvec {\Psi }_{DCT}$, where $\varvec {\Psi }_{DCT}$ sparsifies the spectral-axis while $\varvec {\Psi }_{W2D}$ promotes sparsity along the spatial coordinates. The latter has shown to be a great sparsifying transform for spectral images^18^.

We compared the reconstructions attained with the proposed fusion method against the ones from the MS and HS measurements, independently. The latter are called ‘MS method’ and ‘HS method’, respectively. Although this might seem not fair, given the differences between compression ratios, it permits to demonstrate the fusion of the best of both worlds. Since a ground-truth data cube is not available for comparison, to check the fidelity of the reconstructions, spectral signatures were measured at 4 representative regions of each target scene, highlighted as P1, P2, P3 and P4 in Fig. 4, using a spectrometer (Ocean Optics Flame S-VIS-NIR-ES spectrometer) and assumed to be the ground truth. The root-mean-squared-error (RMSE) and the spectral angle mapper (SAM) were calculated between the reconstructed spectral signatures and the ones measured with the spectrometer. Regarding the proposed ADMM algorithm, two penalization parameters, $\rho$ and $\tau$ in Eqs. (12)–(13), needed to be finely tuned. For this, we followed a cross-validation methodology, varying them within the range $[1e^{-4}, 1]$. In particular, we found that the selection of $\rho =1e^{-2}$ provided the best solution to the $\ell _2-\ell _2$ problem in Eq. (12), independently of the number of snapshots and the method used, but the parameter $\tau$ entailed more sensitive variations in the reconstruction quality, as detailed in Section S4 of the supplementary material.

**Figure 5:**
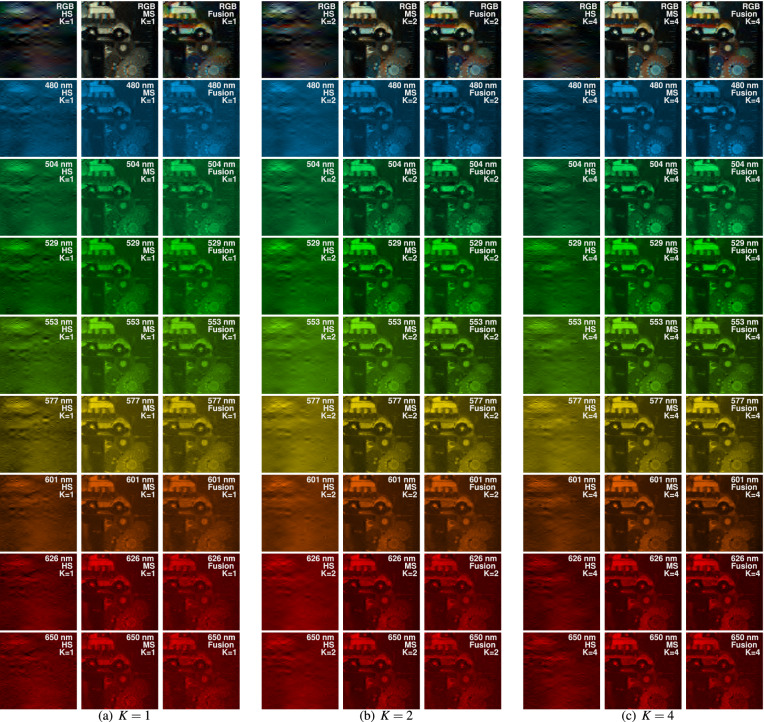
Spectral image reconstructions of the first target scene. From left to right, three columns per snapshot are shown: (**a**) Results for $K=1$, (**b**) Results for $K=2$, and (**c**) Results for $K=4$. From top to bottom, rows show the sRGB-mapped representation of the spectral reconstructions, along with 8 out of the 192 spectral bands. The wavelength, reconstruction method, and the number of snapshots employed are detailed at the top right of each sub-image. This figure was created with Matlab R2019b, https://www.mathworks.com/products/matlab.html, from data acquired with our imaging system.

Figure 5 summarizes the spectral reconstructions of the first target scene using the three different methods, along the three number of snapshots, and fixing the regularization parameters to the best ones for each scenario. In particular, the first row shows the sRGB color-mapped image resulting of mapping the 192 spectral bands to the sRGB spectral sensitivity functions. Rows 2 to 9 present 8 out of the 192 spectral bands, false-colored with the corresponding wavelength (480, 504, 529, 553, 577, 601, 626 and 650 nm). Per row, along the columns, we show three triplets of images corresponding to the three methods using the three different number of snapshots. The first three columns show the MS, HS and fusion results using a single snapshot ($K=1$). Columns 4–6 and 7–9 illustrate the results for 2 and 4 snapshots, respectively. In this figure, it can be first appreciated that the spatial resolution of the reconstruction attained with only the HS measurements, lacks of most of the details of the target scene, whereas the reconstructions achieved with the MS and fusion methods better preserve the spatial structure. Second, the RGB color-mapped images of the HS and fusion reconstructions resemble the color of the target scene better than the MS reconstructions for $K=1$ and $K=2$. These behaviors are expected since the MS measurements lack spectral details, whereas the HS measurements lack spatial details. Third, the reconstructed image quality improves as the number of snapshots increases, independently of the method used. This improvement is appreciated when comparing the HS (columns 1, 4, 7), MS (columns 2, 5, 8), and the fusion (columns 3, 6, 9) reconstructions, separately. Further, although difficult to note, the MS spectra tend to remain constant along contiguous spectral bands, while the HS and fusion reconstructions exhibit noticeable changes between neighboring spectral bands.

**Figure 6:**
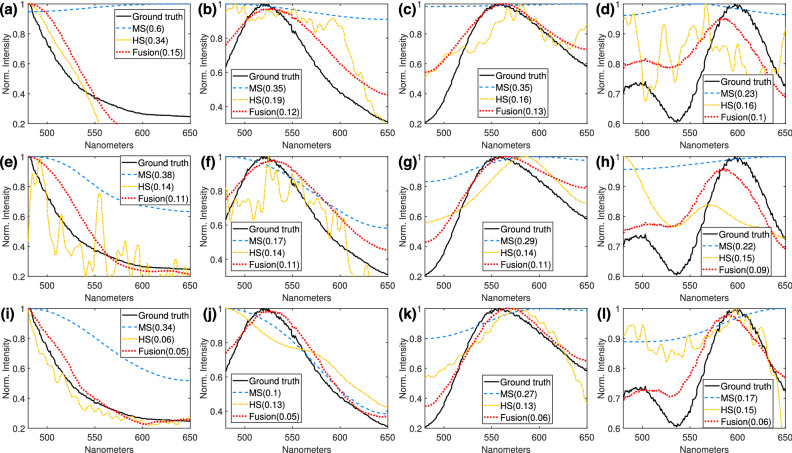
Analysis of the spectral reconstructions of the first target scene. Four different spatial regions (P1, P2, P3, and P4 in Fig. 4a) were measured with a spectrometer and compared against the reconstructed with the different methods and snapshots. (**a**)–(**d**) P1–P4 for $K=1$, (**e**)–(**h**) P1–P4 for $K=2$, (**i**)–(**l**) P1–P4 for $K=4$ snapshots. This figure was created with Matlab R2019b, https://www.mathworks.com/products/matlab.html, from data acquired with our imaging system.

To better appreciate this behavior, the columns of Fig. 6 depicts four representative spectral signatures of the first target scene, measured with the spectrometer (denoted as ground-truth, black solid line), which are compared against the ones reconstructed with the different methods, MS (blue dashed line), HS (yellow dotted line), and fusion (red dash-dotted line). The rows show the results with different number of snapshots. The RMSE for each scenario is detailed in each subfigure legend within parenthesis. Note first, that the spectral signatures better approach the ground-truth as the number of snapshot increases; second, the signatures resulting from the fusion method attain the best fit to the ground-truth; third, the MS signatures are the smoothest and therefore fail in most of the cases (although their spatial extent looks pleasant in Fig. 5); fourth, the HS and fusion signatures are spikier, thus exhibiting better spectral resolution, but the HS signatures look noisier due to the poor spatial quality of their neighborhood.

Results for the second scene are reported in Section S5 of the supplementary material, and an analysis of the time required by the computational algorithm is reported in Section S6 of the supplementary material.

The reconstruction algorithm was programmed in Matlab R2019b, and executed in a desktop computer (details in section S6 of the supplementary material). The stopping criterion was set to be either 500 iterations or until the residual between the updated and previous estimation falls below $10^{-4}$. For the latter, in particular, the algorithm stops when the dual residual $\Vert \kappa (\varvec {\nu }^t -\varvec {\nu }^{(t-1)} )\Vert _2^2 \le 10^{-4}$ and the primal residual $\Vert \varvec {\nu }^t - \varvec {\theta }^t\Vert _2^2 \le 10^{-4}$ with $\kappa = 0.1$. As detailed in Fig. S6, the algorithm’s execution time highly depends on the sparsity-promoting regularization parameter $\tau$. The MS reconstructions are the fastest overall, followed by the fusion and then by the HS reconstructions. Just considering the best regularization parameter for each scenario ($\tau \sim 10^{-1}$), the average execution time lies between 30 and 50 min for the MS reconstructions, 100 to 250 min for the HS, and 80 to 150 min for the fusion methodology, depending on the number of snapshots used. Note that given the high-dimensionality of the final cube ($256\times 256\times 192$), these reconstructions still require hundreds of minutes until convergence. Nonetheless, different optimization algorithms may lead to faster estimation, and future works should undoubtedly look into this matter.

## Discussion

The proposed single aperture compressive snapshot spectral image fusion approach encompasses the following advantages. Given the capabilities of the DMD, pixel binning can be conducted either on the DMD or the sensor pixels, and so, different resolutions can be dynamically attained on the same testbed. More clearly, if a $2\times 2$ binning is conducted on the DMD, the MS measurements can resolve a cube of size $128\times 128\times 4$ and the HS measurements a cube of size $64\times 64\times 96$. On the other hand, binning can also be applied in each sensor independently, thus, different scenarios can be easily tested.

Regarding the improvement entailed by the image fusion method, it should be noted that the MS and HS measurements do not attain a suitable reconstruction, even when the number of snapshots increases. More clearly, the MS measurements alone indeed provide satisfactory spatial quality, but the spectrum remains smooth, whereas on the contrary, the HS measurements yield to better high-resolution signatures, but fail at recovering the spatial structure of the target scenes. With the fusion method we obtain the best of both worlds, that is, good spatial and spectral quality. In this sense, target scenes with sharper spectral signatures (such as light emitting diodes or conventional spectra in related applications such as Raman spectroscopy) would significantly benefit from the improvements attained with the proposed methodology. Moreover, even for slowly-varying spectral signatures the improvement can be appreciated in the results of this work.

It should be highlighted that different compressive image fusion methods have been proposed in the literature^26–29^, but all of them conducted simulations on synthetic data. These papers proposed different reconstruction algorithms, exploiting various data priors such as low-rank and linear mixture models. In contrast, in this paper we imposed just the sparsity regularization constraint, which has been shown to be reliable in the state-of-the-art of CSI experimental testbeds^17,18^. We expect that the different reconstruction and regularization alternatives proposed in the literature will provide further improvements on the results of this manuscript. Nonetheless, these methods are not tested here as we consider the analysis falls out of the current scope.

On the other hand, the proposed imaging system has its own shortcomings, including the difficulty of alignment, the calibration requirements and the bulkiness of the current setup. In particular, given that we exploit the reflecting capabilities of the DMD, the optical trains require precise alignment to correct for the difference in optical path lengths via the Scheimpflug compensation. Furthermore, although not unique for our system, the reconstruction algorithm requires knowledge of the per-pixel PSF; thus, given that we use two sensors with different pixel counts and resolution, mapping to the same DMD, the per-pixel calibration can become tedious, particularly because narrow-band monochromatic beams that match the dispersion functions of both dispersive elements must be used. Finally, the current testbed is bulky, which in turn, limits its usage in outdoor applications. Nonetheless, the first two shortcomings are processes that must be conducted offline and only once, and the last one is still an open challenge for future work.

In conclusion, we have demonstrated spectral image fusion from compressed measurements using a single aperture, snapshot and full-throughput optical testbed. The single aperture capability is advantageous for dual-sensor imaging systems, since it eliminates the requirement of registration, which may cause major problems such as occlusion. The snapshot capability circumvents the need of scanning the scene, which prohibits its usage in dynamic scenes. Finally, the light throughput capability is critical, since coding-based devices undeniably throw away light in the coding step. In this regard, the proposed system exploits the capabilities of the DMDs and takes advantage of the ‘on’ and ‘off’ reflecting properties of the micro-mirrors to implement a full throughput sensing strategy. For the latter, the Scheimpflug compensation was required and successfully implemented. Further, an ADMM-based reconstruction algorithm was proposed to conduct image fusion from the compressed projections, which successfully recovered the fully-resolved spectral image cubes, even for high compression ratios. Future work should undoubtedly focus on the design of optimized coded apertures for image fusion, on the usage of more complex inverse problem formulations to improve image quality, as well as on the miniaturization of the testbed.

## Methods

### Compressive spectral image fusion sensing model

The proposed testbed in Fig. 1b has two propagation paths, split by the DMD, but a single objective lens common to both. In particular, the three-dimensional light field that enters the imaging system can be modeled as $f(x,y,\lambda )$. After the DMD, this field splits into two coded fields, $t(x,y)f(x,y,\lambda )$ and $t^c(x,y)f(x,y,\lambda )$, where *t*(*x*, *y*) is the coding function entailed by the DMD and $t^c(x,y)$ denotes its complement. These coded fields are independently dispersed and integrated at the corresponding MS and HS imaging arms, along their sensor sensitivity functions $\Lambda _M$ and $\Lambda _H$, following,

1 $$\begin{aligned} g_M(x,y)= & {} \int _{\Lambda _M} \iint t(x,y)f(x,y,\lambda )\delta _M(x-x'-d_M(\lambda ),y-y', \lambda ) dx'dy' d\lambda + \eta _M(x,y) \end{aligned}$$

2 $$\begin{aligned}= & {} \int _{\Lambda _M} t(x+d_M(\lambda ),y)f(x+d_M(\lambda ),y,\lambda ) d\lambda + \eta _M(x,y), \end{aligned}$$

where $\delta _M(x,y, \lambda )$ represents the point spread function (PSF) of the system, $d_M(\lambda _{k_M+1}) - d_M(\lambda _{k_M}) = \Delta _M$ models the dispersion introduced by the prism, $\Delta _M$ is the pixel size of the MS sensor, $k_M = 0, \ldots , L_M-1$ indexes the MS wavebands and $\eta _M(x,y)$ accounts for the MS sensor noise. Equation (2) is discretized by the MS sensor at pixel (*m*, *n*) as,

3 $$\begin{aligned} \!\!\!\!\!\! (g_M)_{m,n}= & {} \iint g_M(x,y)\times \text {rect}\left( \frac{x}{\Delta _M}-n, \frac{y}{\Delta _M}-m\right) dxdy \end{aligned}$$

4 $$\begin{aligned}= & {} \iint \left[ \int _{\Lambda _M} t(x+d_M(\lambda ),y)f(x+d_M(\lambda ),y,\lambda ) d\lambda + \eta _M(x,y) \right] \times \text {rect}\left( \frac{x}{\Delta _M}-n, \frac{y}{\Delta _M}-m\right) dxdy \end{aligned}$$

5 $$\begin{aligned}= & {} \displaystyle \sum _{m',n',k_M} \!\! t_{m',n'} f_{m',n'+k_M,k_M}\iint \!\!\left[ \int _{\lambda _{k_M}}^{\lambda _{k_M+1}} \text {rect} \left( \frac{x \!+\! d_M(\lambda )}{\Delta _D} \!-\! n', \frac{y}{\Delta _D} \!-\! m'\right) \times \text {rect}\left( \frac{x}{\Delta _M} \!-\! n, \frac{y}{\Delta _M} \!-\! m\right) d\lambda \right] dxdy \end{aligned}$$

6 $$\begin{aligned}= & {} \displaystyle \sum _{r_M=0}^{R_M-1}\sum _{k_M=0}^{L_M-1} t_{m,n+k_M+r_M} f_{m,n+k_M+r_M,k_M} (w_M)_{k_M}^{r_M} + (\eta _M)_{m,n}, \end{aligned}$$

for $m=0,1,\ldots , M-1$, $n=0,1,\ldots , N+L_M+R_M-2$, assuming the input data has $M\times N$ pixels of spatial resolution, and that $\Delta _D$ is the DMD pixel size. Since 1:1 matching is guaranteed between the DMD and MS sensor pixels after down-sampling, then $\frac{\Delta _D}{\Delta _M}=1$. Equation (5) follows after discretization of the coded aperture, $t(x,y) = \sum _{m',n'}t_{m',n'} \text {rect}(\frac{x}{\Delta _D}-n', \frac{y}{\Delta _D}-m')$, where $t_{m',n'} \in \{ 0, 1 \}$ represents the coding performed by the $(m',n')^{th}$ DMD pixel; and discretization of the spectral data, $f(x,y,\lambda ) = \sum _{m',n',k_M}f_{m',n',k_M} \text {rect}(\frac{x}{\Delta _D}-n', \frac{y}{\Delta _D}-m', \frac{\lambda }{\Delta _M}-k_M)$. Note that, $(w_M)_{k_M}^{r_M}$, with $r_M=0, 1, \ldots , R_M-1$ represents the light distribution and integration along the MS sensor, which accounts for non-linearities and blurring induced by the optics. The latter are the propagation weights calibrated and reported in Table S1 in the supplementary material. Here, $R_M=3$ represents the number of MS sensor pixels affected by a single DMD pixel, as shown in Fig. 3.

In a similar way, the continuous and discrete measurements acquired by the HS imaging arm are given by

7 $$\begin{aligned} \!\!\!\!\!g_H(x,y)= & {} \!\! \int _{\Lambda _H} \!\!\!\!\!\! t^c(x+d_H(\lambda ),y)f(x+d_H(\lambda ),y,\lambda ) d\lambda \!+\! \eta _H(x,y); (g_H)_{i,j} =\!\!\sum _{r_H=0}^{R_H-1}\displaystyle \nonumber \\&\sum _{k_H=0}^{L_H-1} t^c_{i,j+k_H+r_H} f_{i,j+k_H+r_H,k_H} (w_H)_{k_H}^{R_H} + (\eta _H)_{i,j}, \end{aligned}$$

for $i\!=\!0,1,\ldots , I\!-\!1$, $j\!=\!0,1,\ldots , J\!-\!1$, where $I \!=\! \frac{M}{\Delta }$, $J \!=\!\frac{N}{\Delta }$, $\Delta \!=\! \frac{\Delta _M}{\Delta _H}$ and $\Delta _H$ is the pixel size of the HS sensor. Similarly to Eq. (5), $t^c(x,y) = \sum _{m',n'}t^c_{m',n'} \text {rect}(\frac{x}{\Delta _D}-n', \frac{y}{\Delta _D}-m')$, where $t^c_{m',n'} = 1- t_{m',n'}$ represents the complementary coding by the $(m',n')^{th}$ DMD pixel, $d_H(\lambda _{k_H+1}) \!-\! d_H(\lambda _{k_H}) \!=\! \Delta _H$ is the dispersion introduced by the diffraction grating, $k_H \!=\! 0, 1, \ldots , L_H\!-\!1$ indexes the HS wavebands and $(w_H)_{k_H}^{r_H}$ are the propagation weights calibrated and reported in Table S2 of the supplementary material, with $R_H = 9$ based on Fig. 3.

In order to establish a linear system to describe the compressive measurements acquisition, ${\mathbf {f}}$ is defined as a column vector containing $f_{m,n,k_H}$ for the high-resolution spectral components $k_H$ and all high-resolution spatial pixels *m* and *n*. Assuming a coding element with $M \times N$ pixels, ${\mathbf {f}}$ will be $MNL_H$ in length. Then, define ${\mathbf {g}}_M$ and ${\mathbf {g}}_H$ as column vectors holding the recorded pixel values, $(g_M)_{m,n}$ and $(g_H)_{i,j}$, from the multispectral and hyperspectral image sensors. Noting the horizontal dispersion of light, and the different dispersive elements used by the multispectral and hyperspectral imaging arms, the length of ${\mathbf {g}}_M$ is $M (N + L_M - 1)$, and ${\mathbf {g}}_H$ is $I (J + L_H - 1)$, such that all light is accounted for on the sensors. Therefore, the proposed image acquisition system can be modeled according to the linear equations,

8 $$\begin{aligned} {\mathbf {g}}_M = {\mathbf {H}}_M {\mathbf {f}} + \varvec {\eta }_M = {\mathbf {P}}_M {\mathbf {D}}_M {\mathbf {T f}} + \varvec {\eta }_M, {\mathbf {g}}_H = {\mathbf {H}}_H {\mathbf {f}} + \varvec {\eta }_H = {\mathbf {D}}_H {\mathbf {P}}_H {\mathbf {T}}^c{\mathbf {f}} + \varvec {\eta }_H, \end{aligned}$$

where ${\mathbf {D}}_M$ and ${\mathbf {D}}_H$ model the spectral and spatial down-sampling operators, respectively. The dispersion function of the prism is modeled as ${\mathbf {P}}_M$, and the matrix ${\mathbf {P}}_H$ denotes that of the grating. Note that $\varvec {\eta }_M$ and $\varvec {\eta }_H$ model the additive noise terms in both sensors.

### Fusion+reconstruction algorithm

Having modeled the collection of incoming light rays per sensor, the measurements are assembled into a single linear system by vertically concatenating ${\mathbf {g}}_M$ and ${\mathbf {g}}_H$, to create the $(M(N+L_M-1) + I(J+L_H-1))\times 1$ vector ${\mathbf {g}} = \left[ {\mathbf {g}}_M^T, {\mathbf {g}}_H^T\right] ^T$, which can be modeled by similarly concatenating the matrices ${\mathbf {H}}_M$ and ${\mathbf {H}}_H$ on top of one another, ${\mathbf {H}} = \left[ ({\mathbf {P}}_M {\mathbf {D}}_M {\mathbf {T}})^T, ({\mathbf {D}}_H {\mathbf {P}}_H {\mathbf {T}}^c)^T \right] ^T$, so as to obtain the compact linear system, ${\mathbf {g}} = {\mathbf {H}}{\mathbf {f}}+\varvec {\eta }$, where $\varvec {\eta } = \left[ \varvec {\eta }_M^T, \varvec {\eta }_H^T\right] ^T$. The compression ratio of the proposed architecture can be defined as, $C = 1 - K(\frac{M(N+L_M-1) + I(J+L_H-1)}{MNL_H})$, where *K* is the number of snapshots acquired in each sensor. Note that $C \in \left[ 0, 1\right]$, with $C=0$ means no compression and $C=1$ indicates a compression of $100\%$. Note also that *C* reduces with the increasing number of snapshots. Thus, each new snapshot, employing a different coded aperture, yields a reduction in compression and therefore helps to relax the conditioning of the inverse problem. If multiple snapshots are acquired, they are vertically concatenated as ${\mathbf {g}} = \left[ {\mathbf {g}}_0^T, {\mathbf {g}}_1^T, \ldots , {\mathbf {g}}_{K-1}^T \right] ^T$ and the sensing matrix becomes ${\mathbf {H}} = \left[ {\mathbf {H}}_0^T, {\mathbf {H}}_1^T, \ldots , {\mathbf {H}}_{K-1}^T \right] ^T$. To reconstruct a fused HS image with high-spatial and high-spectral resolution from the compressed measurements, we propose to solve,

9 $$\begin{aligned} {\mathbf {f}} = \varvec {\Psi } ( \underset{\varvec {\theta }}{\text {argmin}} \left\{ \Vert {\mathbf {g}} - \mathbf {H}\varvec {\Psi \theta } \Vert _2^2 + \tau \Vert \varvec {\theta }\Vert _1 \right\} ), \end{aligned}$$

which assumes that $\mathbf {f} = \varvec {\Psi \theta }$, where $\varvec {\Psi }$ is a transform basis that sparsifies the HS image. This formulation finds a sparse solution to the unconstrained minimization problem in Eq. (9), where the first term minimizes the Euclidean distance between the compressed measurements and the contribution from the estimate $\varvec {\theta }$, while the second term encourages sparsity of the reconstruction, and $\tau$ is a tuning parameter that controls the extent of smoothness in the estimate. Due to the huge size of the data, Eq. (9) is rewritten as a quadratic program through an alternating direction method of multipliers (ADMM) formulation, such that smaller sub-problems can be solved. In particular, the unconstrained formulation becomes the constrained problem,

10 $$\begin{aligned} \underset{\varvec {\theta }}{\text {argmin}} \left\{ \Vert \mathbf {g} - \mathbf {H}\varvec {\Psi }\varvec {\nu } \Vert _2^2 + \tau \Vert \varvec {\theta }\Vert _1 \right\} , \text {subject to} \varvec {\nu }= \varvec {\theta }, \end{aligned}$$

where $\varvec {\nu }$ is an auxiliary variable. The augmented Lagrangian of Eq. (10) is given by,

11 $$\begin{aligned} \mathcal {L}(\varvec {\theta }, \mathbf {d}, \varvec {\nu }) = \Vert \mathbf {g} - \mathbf {H}\varvec {\Psi }\varvec {\nu } \Vert _2^2 + \tau \Vert \varvec {\theta }\Vert _1 + \rho \Vert \varvec {\nu } - \varvec {\theta } - \mathbf {d}\Vert _2^2, \end{aligned}$$

where $\mathbf {d}$ is the scaled dual variable and $\rho >0$ is the weighting of the augmented Lagrangian term. Based on Eq. (11), the alternative form of Eq. (9) can be expressed as the following two optimization problems, plus the dual variable update,

12 $$\begin{aligned} \varvec {\nu }^{q+1}= & {} \underset{\varvec {\nu }}{\text {argmin}} \left( \Vert \mathbf {g} - \mathbf {H}\varvec {\Psi }\varvec {\nu }^{q} \Vert _2^2 + \rho \Vert \varvec {\nu }^{q} - \varvec {\theta }^{q} - \mathbf {d}^{q}\Vert _2^2 \right) , \end{aligned}$$

13 $$\begin{aligned} \varvec {\theta }^{q+1}= & {} \underset{\varvec {\theta }}{\text {argmin}} \left( \rho \Vert \varvec {\nu }^{q+1} - \varvec {\theta }^{q} - \mathbf {d}^{q}\Vert _2^2 + \tau \Vert \varvec {\nu }^{q+1}\Vert _1 \right) , \end{aligned}$$

14 $$\begin{aligned} \mathbf {d}^{q+1}= & {} \mathbf {d}^{q} + \rho (\varvec {\nu }^{q+1}-\varvec {\theta }^{q+1}), \end{aligned}$$

for $q=0, \ldots , Q-1$, where *Q* is the maximum number of iterations of the algorithm. To solve Eq. (12) we derive with respect to $\varvec {\nu }$ and find its closed solution,

15 $$\begin{aligned} \varvec {\nu }^{q+1} = \left[ \mathbf {A}^T\mathbf {A} + \rho \mathbf {I}\right] ^{-1} \left[ \mathbf {A}^T\mathbf {g} + \rho (\varvec {\theta }^{q} + \mathbf {d}^q) \right] = \left[ \frac{1}{\rho }\mathbf {I} -\frac{1}{\rho } \mathbf {A}^T \left( \mathbf {I} + \frac{1}{\rho } \mathbf {H}\mathbf {H}^T \right) ^{-1}\mathbf {A}\right] \left[ \mathbf {A}^T\mathbf {g} + \rho (\varvec {\theta }^q + \mathbf {d}^q) \right] , \end{aligned}$$

where $\mathbf {A}=\mathbf {H}\varvec {\Psi }$ and the right hand of Eq. (15) is obtained after applying the Sherman–Morrison–Woodbury matrix inversion lemma^35^. The latter is critical since the matrix inversions at the left-hand side of the second equality depend on the size of the high-spatial and high-spectral resolution image, whereas the Woodbury lemma transforms these inversions to depend on the size of the compressed measurements, which is at least $L_H$ times smaller. On the other hand, to solve Eq. (13), we calculate its derivative with respect to $\varvec {\theta }$ and find its closed-form solution, which leads to the soft-thresholding operator,

16 $$\begin{aligned} \varvec {\theta }^{q+1} = \text {soft}\left( \varvec {\theta }^q+\mathbf {d}^q, \tau /\rho \right) = \max (|\varvec {\theta }^q+\mathbf {d}^q | - \tau /\rho , 0)\text {sign}(\varvec {\theta }^q+\mathbf {d}^q). \end{aligned}$$

The algorithm iterates throughout Eqs. (12)–(16) until reaching a maximum number of iterations or certain error tolerance.

## Supplementary Information

Supplementary Information.

## Data Availability

The datasets generated and/or analyzed during the current study are available from the corresponding author on request.

## References

[1] Kruse FA, Boardman JW, Huntington JF (2003). Comparison of airborne hyperspectral data and eo-1 hyperion for mineral mapping. IEEE Trans. Geosci. Remote Sens..

[2] Bioucas-Dias JM (2013). Hyperspectral remote sensing data analysis and future challenges. IEEE Geosci. Remote Sens. Mag..

[3] Sellar, R. G. & Boreman, G. D. Classification of imaging spectrometers for remote sensing applications. *Opt. Eng.* **44**, 44 (2005).

[4] Lu, G. & Fei, B. Medical hyperspectral imaging: a review. *J. Biomed. Opt.* **19**, 1–23 (2014). 10.1117/1.JBO.19.1.010901 PMC3895860 24441941

[5] Schultz RA (2001). Hyperspectral imaging: a novel approach for microscopic analysis. Cytometry.

[6] Levenson RM, Mansfield JR (2006). Multispectral imaging in biology and medicine: slices of life. Cytom. Part A.

[7] Eismann, M. *Hyperspectral Remote Sensing* (SPIE, SPIE Press, 2012).

[8] Schueler, C. F. Image quality vs. sensitivity: fundamental sensor system engineering. In Ardanuy, P. E. & Puschell, J. J. (eds.) *Remote Sensing System Engineering*, vol. 7087, 98 – 108. International Society for Optics and Photonics (SPIE, 2008).

[9] Loncan L (2015). Hyperspectral pansharpening: a review. IEEE Geosci. Remote Sens. Mag..

[10] Wei Q, Dobigeon N, Tourneret J, Bioucas-Dias J, Godsill S (2016). R-fuse: robust fast fusion of multiband images based on solving a sylvester equation. IEEE Signal Process. Lett..

[11] Wei Q (2016). Multiband image fusion based on spectral unmixing. IEEE Trans. Geosci. Remote Sens..

[12] Wei Q, Bioucas-Dias J, Dobigeon N, Tourneret J (2015). Hyperspectral and multispectral image fusion based on a sparse representation. IEEE Trans. Geosci. Remote Sens..

[13] Hagen, N., Kester, R. T., Gao, L. & Tkaczyk, T. S. Snapshot advantage: a review of the light collection improvement for parallel high-dimensional measurement systems. *Opt. Eng.* **51**, 111702–1–111702–7 (2012). 10.1117/1.OE.51.11.111702 PMC3393130 22791926

[14] Hagen N, Kudenov MW (2013). Review of snapshot spectral imaging technologies. Opt. Eng..

[15] Brady, D. J. *Optical Imaging and Spectroscopy* (Wiley, 2009).

[16] Arce GR, Brady DJ, Carin L, Arguello H, Kittle DS (2014). Compressive coded aperture spectral imaging: an introduction. IEEE Signal Process. Mag..

[17] Cao X (2016). Computational snapshot multispectral cameras: toward dynamic capture of the spectral world. IEEE Signal Process. Mag..

[18] Arce, G. R., Rueda, H., Correa, C. V., Ramirez, A. & Arguello, H. *Snapshot Compressive Multispectral Cameras*, 1–22 (Wiley, 2017).

[19] August I, Oiknine Y, AbuLeil M, Abdulhalim I, Stern A (2016). Miniature compressive ultra-spectral imaging system utilizing a single liquid crystal phase retarder. Sci. Rep..

[20] Wu, J. *et al.* Snapshot hyperspectral volumetric microscopy. *Sci. Rep.* **6**, 24624 (2016). 10.1038/srep24624 PMC4840377 27103155

[21] Bian L (2016). Multispectral imaging using a single bucket detector. Sci. Rep..

[22] Li Z (2017). Efficient single-pixel multispectral imaging via non-mechanical spatio-spectral modulation. Sci. Rep..

[23] Wang L, Xiong Z, Shi G, Wu F, Zeng W (2016). Adaptive nonlocal sparse representation for dual-camera compressive hyperspectral imaging. IEEE Trans. Pattern Anal. Mach. Intell..

[24] Shmilovich, S. *et al.* Dual-camera design for hyperspectral and panchromatic imaging, using a wedge shaped liquid crystal as a spectral multiplexer. *Sci. Rep.* **10**, 3455 (2020). 10.1038/s41598-020-60413-8 PMC7044303 32103101

[25] Yuan X (2015). Compressive hyperspectral imaging with side information. IEEE J. Sel. Top. Signal Process..

[26] Vargas E, Espitia O, Arguello H, Tourneret J (2019). Spectral image fusion from compressive measurements. IEEE Trans. Image Process..

[27] Vargas E, Arguello H, Tourneret J (2019). Spectral image fusion from compressive measurements using spectral unmixing and a sparse representation of abundance maps. IEEE Trans. Geosci. Remote Sens..

[28] Ramirez JM, Arguello H (2019). Multiresolution compressive feature fusion for spectral image classification. IEEE Trans. Geosci. Remote Sens..

[29] Gelvez, T. & Arguello, H. Nonlocal low-rank abundance prior for compressive spectral image fusion. *IEEE Trans. Geosci. Remote Sens.* **1–11**, (2020).

[30] Maruccio G, Sun C, Liu H, Jia M, Chen S (2018). Review of calibration methods for scheimpflug camera. J. Sens..

[31] Smith, W. J. *Modern Optical Engineering* 4th edn. (McGraw Hill, 2008).

[32] Shepard, R. H. *et al.* Optical design and characterization of an advanced computational imaging system. In Awwal, A. A. S., Iftekharuddin, K. M., Matin, M. A. & Márquez, A. (eds.) *Optics and Photonics for Information Processing VIII*, vol. 9216, 73 – 87. International Society for Optics and Photonics (SPIE, 2014).

[33] Instruments, T. Introduction to +/- 12 degree orthogonal digital micromirror devices (dmds) (2008). https://www.ti.com/lit/an/dlpa008b/dlpa008b.pdf. Accessed: Aug 2020.

[34] Arguello H, Rueda H, Wu Y, Prather DW, Arce GR (2013). Higher-order computational model for coded aperture spectral imaging. Appl. Opt..

[35] Akgün MA, Garcelon JH, Haftka RT (2001). Fast exact linear and non-linear structural reanalysis and the Sherman–Morrison–Woodbury formulas. Int. J. Numer. Methods Eng..

