# Supplementary material for: Compressive spectral image fusion via a single aperture high throughput imaging system
Source: Sci Rep. 2021 May 13;11:10311. doi: 10.1038/s41598-021-89788-y (PMC8119686; doi:10.1038/s41598-021-89788-y)
Supplement: Supplementary file 1 — Supplementary Information. [file 41598_2021_89788_MOESM1_ESM.pdf]

# Supplementary material for paper: Compressive Spectral Image Fusion via a Single Aperture High Throughput Imaging System

**Hoover Rueda-Chacon, Fernando Rojas, Henry Arguello**

Department of Computer Science, Universidad Industrial de Santander, Bucaramanga, 680002, Colombia

## S1 Technical details of the Scheimpflug compensation

Remark that the Scheimpflug compensation is required due to the use of a DMD to attain a full-throughput imaging system. State-of-the-art CSI systems typically employ coded apertures, implemented via photomasks, which causes losses in light intensity because the blocked light is not permitted inside the imaging system. In contrast, in our proposed imaging system the coded aperture is implemented via a DMD instead of a photomask, such that it does not block the light, but reflects it onto two different angles (+24° and -24°). Therefore, to guarantee full light throughput, it is required to precisely align the two imaging systems (MS and HS imaging arms) along these DMD angles. Given that the light intensity from the scene impinges the DMD perpendicularly, the rotation angle of the micromirrors causes an optical path length difference (OPLD) between the borders of each micromirror, therefore yielding a defocused-and-distorted image at the sensor plane, which is located parallel to the DMD, as it is depicted in Fig. S1. To correct for this OPLD, the sensor plane must be rotated proportionally to the micromirror angle, to guarantee that light from the scene plane travels the exact same distance until the sensor plane. This correction angle has been coined Scheimpflug angle, and it is mathematically denoted as $\varphi$. As stated in the manuscript, for the case of the MS imaging arm, this angle can be calculated via $\varphi_{MS} = \arctan((u'/u)\tan\theta)$, where $u$ and $u'$ are the distances from the DMD to the lens, and from the lens to the sensor, respectively. With this correction, so-called Scheimpflug compensation, the image attained in the sensor is sharp and does no contain distortions, as required to acquire the compressed measurements.

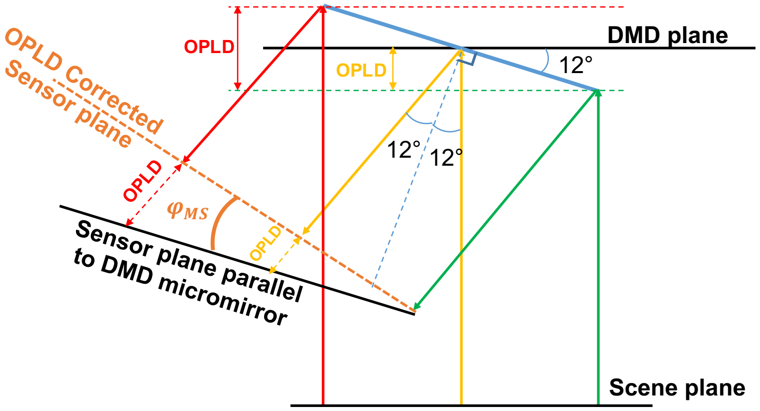


**Supplementary Figure S1. Sketch of the optical path length difference (OPLD) caused by the micromirrors rotation angle.** The sensor plane must be rotated $\varphi$ degrees to compensate for the OPLD, such that all the light rays emanating from the scene plane travel the exact same distance until the sensor plane. These compensation is so-called Scheimplug compensation.

## S2 Details of the proposed testbed setup

Figure S2 shows further details of the proposed testbed setup, including a top view and a back view image of the optical train. In particular, the top view photo highlights the focal lengths of the lenses, the reflection angles of the DMD, the diffraction grating blazing angle as well as the Scheimpflug angle corrections conducted on the image sensors. The back view image is included to better appreciate the accommodation of the different optical elements within the optical train.

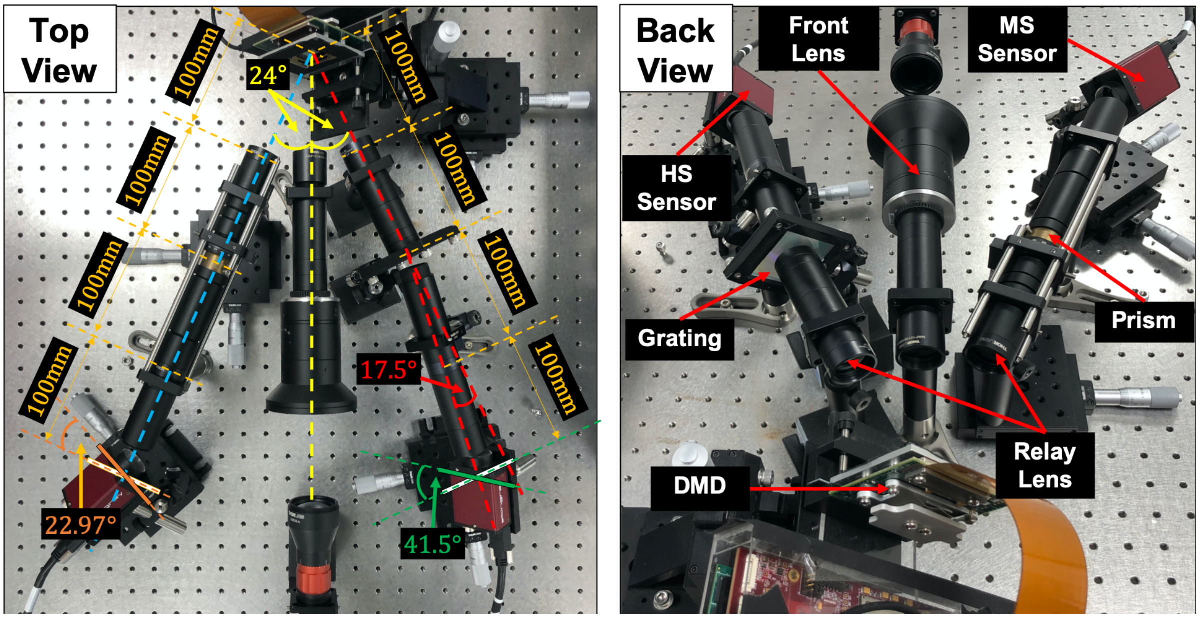


**Supplementary Figure S2. Top view and back view of the proposed testbed.** The front lens images the target scene onto the DMD, which encodes and reflects the scene towards the MS and HS sensors at +/-24°, respectively. The MS and HS imaging arms employ 4F relay lenses, with an effective focal length of 100 mm, to transmit the encoded light onto the sensors, either through the Amici prism, or through the diffraction grating. Note that the MS and HS image sensors are rotated $\varphi_{MS} \approx 22.97°$ and $\varphi_{HS} \approx 41.5°$, respectively, with respect to the original propagation axis.

## S3 Calibration weights of the light propagation through the testbed

The point spread function of the system is calibrated to characterize the light propagation throughout the system. This process is conducted on a per-pixel basis to obtain accurate modeling. Table S1 shows the calibration weights of the MS imaging arm, which correspond to the nonlinear dispersion curve of the Amici prism. This table has 3 rows and 8 columns corresponding to the 3 MS sensor pixels affected by a single pixel of the DMD along the 8 spectral bands that can be reconstructed with the MS imaging arm. Similarly, Table S2 includes the calibration weights of the HS imaging arm, which correspond to the linear dispersion curve of the diffraction grating. This table has 9 rows and 192 columns representing the 9 HS sensor pixels where light from each DMD pixel impinges along the 192 spectral bands that the HS arm can resolve.

| Weight | $k_M = 0$ | $k_M = 1$ | $k_M = 2$ | $k_M = 3$ | $k_M = 4$ | $k_M = 5$ | $k_M = 6$ | $k_M = 7$ |
|---|---|---|---|---|---|---|---|---|
| $(w_M)^0_{k_M}$ | 0.16 | 0.15 | 0.15 | 0.14 | 0.13 | 0.13 | 0.12 | 0.12 |
| $(w_M)^1_{k_M}$ | 0.63 | 0.66 | 0.67 | 0.69 | 0.71 | 0.71 | 0.74 | 0.76 |
| $(w_M)^2_{k_M}$ | 0.21 | 0.19 | 0.18 | 0.17 | 0.16 | 0.16 | 0.14 | 0.12 |

**Table S1.** Multispectral dispersion weights distribution

| Weight | $k_H = 0$ | $k_H = 1$ | $k_H = 2$ | $\cdots$ | $k_H = 191$ |
|---|---|---|---|---|---|
| $(w_H)^0_{k_H}$ | 0.02 | 0.02 | 0.02 | $\cdots$ | 0.02 |
| $(w_H)^1_{k_H}$ | 0.05 | 0.06 | 0.05 | $\cdots$ | 0.06 |
| $(w_H)^2_{k_H}$ | 0.10 | 0.12 | 0.10 | $\cdots$ | 0.10 |
| $(w_H)^3_{k_H}$ | 0.15 | 0.17 | 0.15 | $\cdots$ | 0.15 |
| $(w_H)^4_{k_H}$ | 0.18 | 0.19 | 0.19 | $\cdots$ | 0.18 |
| $(w_H)^5_{k_H}$ | 0.18 | 0.17 | 0.18 | $\cdots$ | 0.18 |
| $(w_H)^6_{k_H}$ | 0.14 | 0.12 | 0.14 | $\cdots$ | 0.14 |
| $(w_H)^7_{k_H}$ | 0.10 | 0.10 | 0.11 | $\cdots$ | 0.11 |
| $(w_H)^8_{k_H}$ | 0.08 | 0.05 | 0.06 | $\cdots$ | 0.06 |

**Table S2.** Hyperspectral dispersion weights

## S4 Cross-validation of the sparsity-promoting regularization parameter $\tau$

In this section, we show the results of the cross-validation of the penalization parameter $\tau$ in Eq. (13), which needed to be finely tuned. The cross-validation methodology evaluates this parameter within the range between $[1e^{-4}, 1]$. Figure S3 depicts the variability of the reconstruction performance, measured in terms of the RMSE and SAM between the ground truth spectral signatures measured with the spectrometer and the same spots from the reconstructions, for the three different methods, MS

(blue dashed line), HS (yellow dotted line), Fusion (red dash-dotted line), along the different $\tau$ values. Note that the best $\tau$ depends on the method used as well as on the number of snapshots employed. To perform a fair comparison, we selected the best parameters for each scenario.

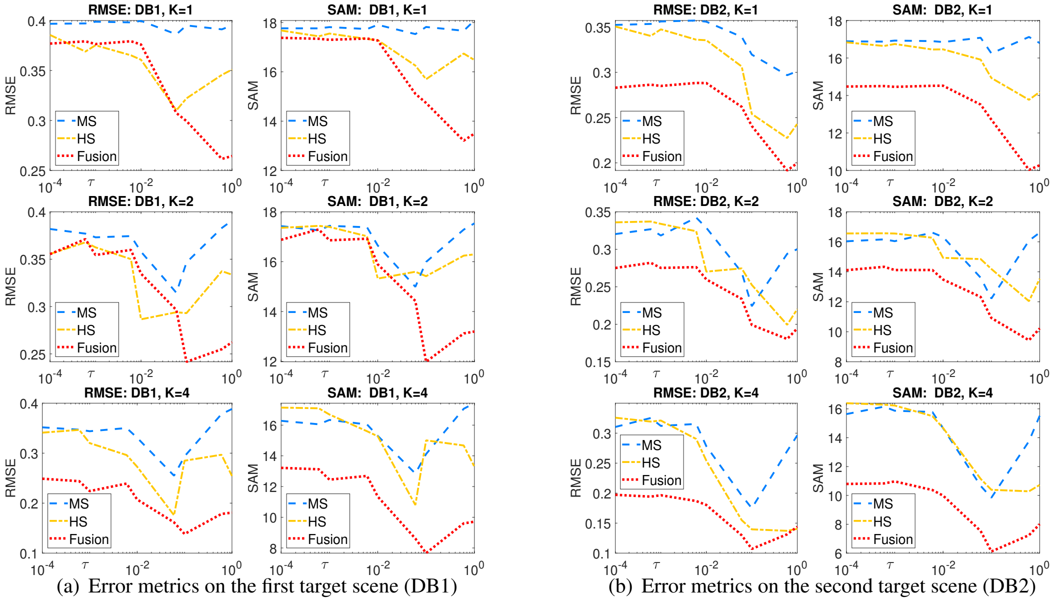


(a) Error metrics on the first target scene (DB1) (b) Error metrics on the second target scene (DB2)

**Supplementary Figure S3. Cross-validation analysis of the reconstruction quality in terms of the sparsity promoting penalization parameter $\tau$.** (a) RMSE and SAM of the first target scene (DB1). (b) RMSE and SAM of the second target scene (DB2). Top-to-bottom rows show the results varying the number of snapshots ($K$) between 1, 2 and 4, respectively. These error metrics were calculated between the ground truth spectral signatures (P1-P4 in Fig. 4) measured with the spectrometer and those attained with the reconstructions, and then averaged. This figure was created with Matlab R2019b, https://www.mathworks.com/products/matlab.html, from data acquired with our imaging system.

## S5 Fusion+Reconstruction results of the second target scene

Reconstruction results for the second target scene are reported in this subsection. This target scene exhibits a high detailed background, with horizontal stripes proper of the fabric where stars are printed. Contrarily to the first scene, no dark spots are present in the second scene. This can be considered as a more challenging scene. Reconstructions at monochromatic level are detailed in Fig. S4. In particular, in this figure we can appreciate and confirm that increasing the number of snapshots improves the system conditioning and then provides better image quality. Further, the HS reconstructions exhibit poor spatial quality, and a larger number of snapshots is not enough to improve it. Regarding the MS reconstructions, it can be seen that colors vanish for a single snapshot, since an almost grayscale image is appreciated in the RGB-mapped image, but it greatly improves for $K = 2$ and $K = 4$. Finally, the fused reconstructions exhibit a comparable spatial quality to the MS results, but with better vibrant color representation, easily noted when a single snapshot is employed. The latter is expected from the better spectral resolution provided by the fusion method. Note that the challenging striped background is hardly appreciated in the $K = 1$ and $K = 2$ reconstructions, but it is better defined on the $K = 4$ results.

To better appreciate the improvement in spectral reconstruction quality, the four spectral signatures, denoted as P1-P4 in Fig. 4(b), were measured with a spectrometer, plotted in Fig. S5 as the ground-truth (solid black line), and compared against the signatures attained in the reconstructions. In this figure it can be appreciated that the signatures reconstructed with the MS measurements (blue dashed lines) are the smoothest overall, and although they adjust better with an increasing number of snapshots, they fail approaching to the curly spectral signatures (P4, shown in the fourth column). On the contrary, the HS signatures (yellow dotted lines) exhibit a higher resolution (oscillatory behavior), but they fail in fitting the ground-truth due to the noisy neighbors (poor spatial resolution). Regarding the spectral signatures of the fusion method, we can appreciate the higher spectral resolution as in the HS results, but less oscillatory, which can be associated with the equilibrium the MS measurement imposes to the fused reconstruction. Overall, the fusion method entails the best fitting to the ground-truth spectral signatures.

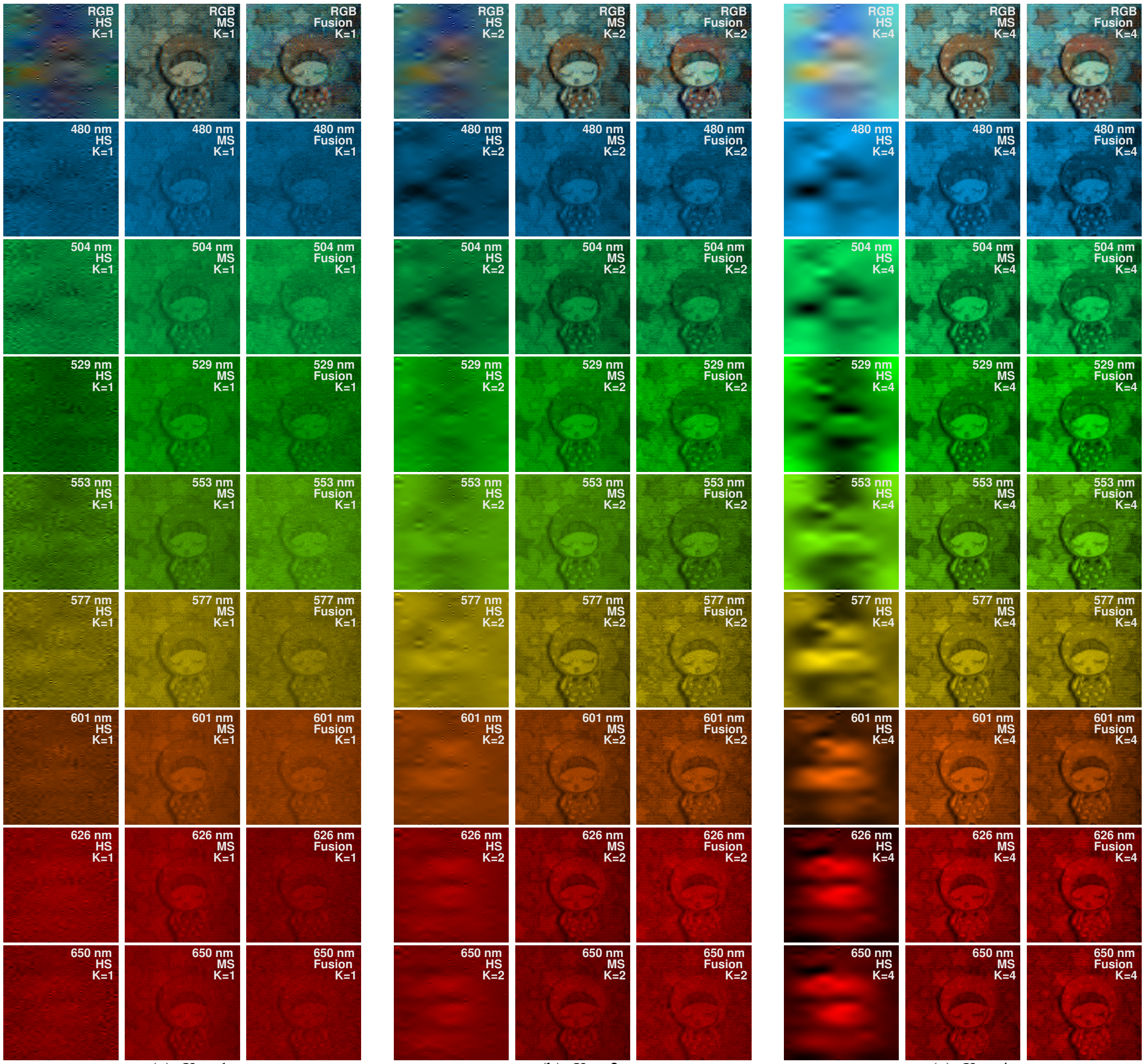


(a) $K = 1$ (b) $K = 2$ (c) $K = 4$

**Supplementary Figure S4. Spectral image reconstructions of the second target scene.** From left to right, three columns per snapshot are shown: (a) Results for $K = 1$, (b) Results for $K = 2$, and (c) Results for $K = 4$. From top to bottom, rows show the sRGB-mapped representation of the spectral reconstructions, along with 8 out of the 192 spectral bands. The wavelength, reconstruction method, and the number of snapshots employed are detailed at the top right of each sub-image. This figure was created with Matlab R2019b, `https://www.mathworks.com/products/matlab.html`, from data acquired with our imaging system.

## S6 Time required by the image fusion+reconstruction algorithm

We evaluated the time required by the proposed algorithm to conduct the fusion and reconstruction. All the reconstructions were conducted and timed using an Intel(R) Core(TM) i7-4790 @ 3.60 GHz processor with 32 GB of DDR3 RAM (4 DIMM of 8 GB each); no acceleration or parallel processing was used. We measured the time at the different cross-validated values of $\tau$. Figure S6 summarizes the execution time of the algorithm, measured in minutes, for the different methods used and the amount of snapshots employed. In this figure we can appreciate that the execution time highly depends on the sparsity-promoting regularization parameter. The MS method shows to be the fastest overall, followed by the fusion method and lastly by the HS method. Remark that the best regularization parameter tends to be in the order of $10^{-1}$, as shown in Fig. S3. Thus, the average execution time at $10^{-1}$ lies between 30 to 50 minutes for the MS method, 100 to 250 minutes for the HS method and 80 to 150 minutes for the fusion methodology.

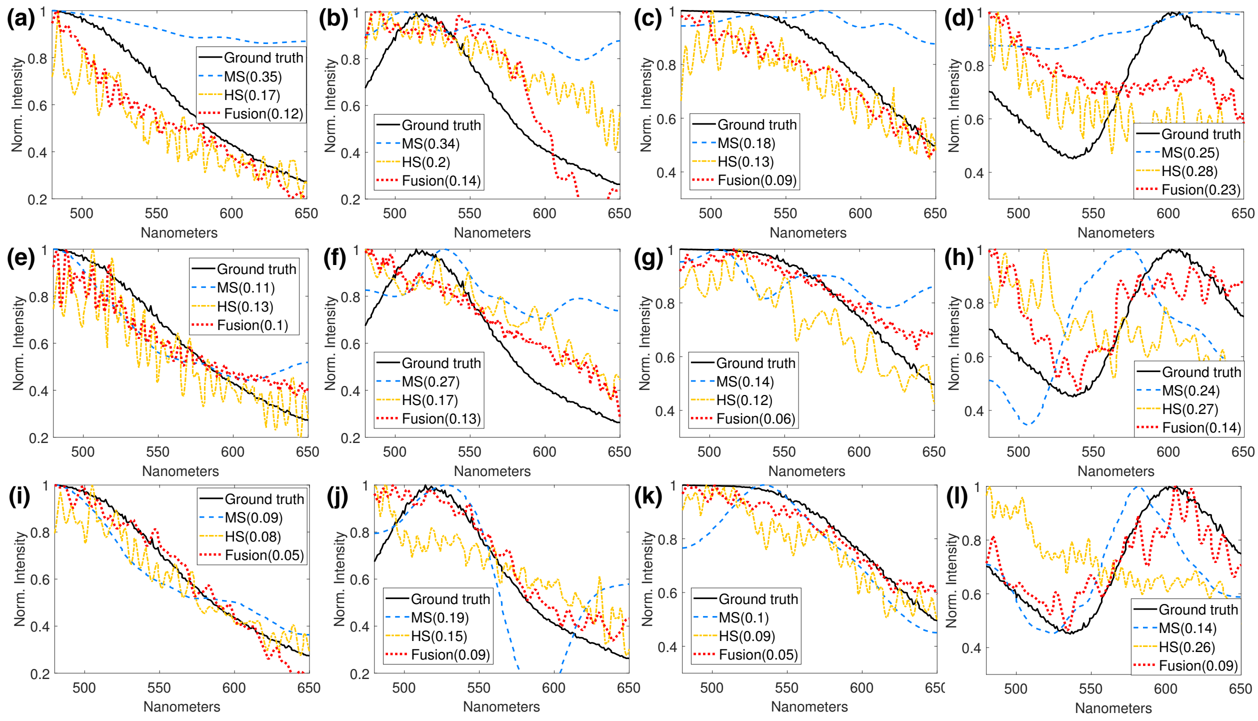


**Supplementary Figure S5. Analysis of the spectral reconstructions of the second target scene.** Four different spatial regions (P1, P2, P3, and P4 in Fig. 4(b)) were measured with a spectrometer and compared against the reconstructed with the different methods and snapshots. (a)-(d) P1-P4 for $K = 1$, (e)-(h) P1-P4 for $K = 2$, (i)-(l) P1-P4 for $K = 4$ snapshots. This figure was created with Matlab R2019b, `https://www.mathworks.com/products/matlab.html`, from data acquired with our imaging system.

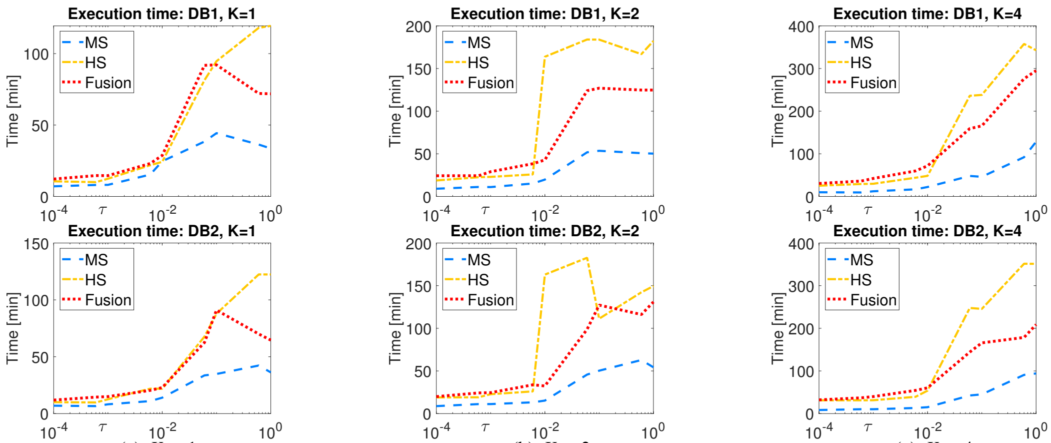


(a) $K = 1$ (b) $K = 2$ (c) $K = 4$

**Supplementary Figure S6. Cross-validation analysis of the time required by the algorithm until convergence, in terms of the sparsity-promoting penalization parameter $\tau$.** Execution time for the first and second target scene using: (a) $K = 1$. (b) $K = 2$, and (c) $K = 4$ snapshots, respectively. The first row shows the results for the first target scene (DB1), and the second row for the second target scene (DB2). This figure was created with Matlab R2019b, `https://www.mathworks.com/products/matlab.html`, from data acquired with our imaging system.
